# Ubiquitin-Dependent Regulation of Influenza A Virus Polymerase and vRNP Function: Mechanisms and Therapeutic Opportunities

**DOI:** 10.3390/microorganisms14081684

**Published:** 2026-07-31

**Authors:** Ren Cao, Feng Guo, Ting Huang, Yuxuan Zhang, You Chen, Jinwei Yuan, Tianyang Fu, Zhongfang Wang, Donglan Liu

**Affiliations:** 1 Guangzhou National Laboratory, Guangzhou International Bio-Island, Guangzhou 510320, China; 2024311458@stu.gzhmu.edu.cn (R.C.); 18218565826@163.com (Y.C.); yuanjinwei2020@163.com (J.Y.); 2022125016@stu.gzhmu.edu.cn (T.F.); 2 Jiangsu Key Laboratory of Immunity and Metabolism, Department of Pathogenic Biology and Immunology, Xuzhou Basic Medical School, Xuzhou Medical University, Xuzhou 221004, China; feng.guo@xzhmu.edu.cn; 3 State Key Laboratory of Respiratory Disease, National Clinical Research Center for Respiratory Disease, Guangzhou Institute of Respiratory Health, the First Affiliated Hospital of Guangzhou Medical University, Guangzhou 510120, China; huangting1161@163.com (T.H.); zyxmanzyxman@outlook.com (Y.Z.); 4 School of Pharmaceutical Sciences, Shenzhen Campus of Sun Yat-sen University, Shenzhen 518107, China

**Keywords:** influenza A virus, RNA-dependent RNA polymerase, polymerase activity, post-translational modification, ubiquitination, deubiquitinase, host-directed therapies

## Abstract

Influenza A virus (IAV) remains a major threat to global public health because of its capacity for antigenic drift, reassortment, zoonotic transmission, and pandemic emergence. Viral transcription and genome replication are carried out by the influenza virus RNA-dependent RNA polymerase (FluPol), a heterotrimeric complex composed of polymerase basic protein 1 (PB1), polymerase basic protein 2 (PB2), and polymerase acidic protein (PA), which functions together with nucleoprotein (NP) within viral ribonucleoprotein complexes (vRNPs). FluPol activity is regulated not only by viral determinants and host cofactors but also by diverse post-translational modifications. Among these, ubiquitination has emerged as a particularly versatile regulatory mechanism because it can control protein stability, polymerase assembly, subunit interactions, conformational dynamics, NP–RNA interactions, and innate immune signaling. Depending on the modified substrate, ubiquitin linkage type, acceptor residue, and responsible E3 ligase or deubiquitinase, ubiquitination may either restrict IAV replication or be exploited by the virus to enhance polymerase function and vRNP activity. This review summarizes recent advances in ubiquitination-mediated regulation of FluPol and NP, focusing on the responsible E3 ubiquitin ligases, deubiquitinases, ubiquitination sites, ubiquitin-chain types, and host restriction mechanisms. We further discuss the crosstalk between ubiquitination and other post-translational modifications, highlight unresolved mechanistic questions, and evaluate the therapeutic potential and challenges of targeting ubiquitin-dependent pathways for antiviral intervention. Collectively, this review provides a conceptual framework for understanding how ubiquitination shapes IAV replication and identifies E3 ligases and DUBs as potential targets for host-directed antiviral strategies.

## 1. Introduction

IAV remains a major global public health concern, causing recurrent seasonal epidemics and occasional pandemics associated with substantial morbidity and mortality [1,2]. Its segmented, negative-sense RNA genomes are subject to frequent mutation and reassortment, which drive antigenic drift and antigenic shift and facilitate viral adaptation to new hosts as well as the emergence of zoonotic or pandemic strains [3,4,5]. IAV circulates widely among avian and mammalian hosts, including wild birds, poultry, swine, and humans. Several avian subtypes, such as H5N1, H5N6, H7N9, and H9N2, have repeatedly caused zoonotic infections, among which H5N1 and H7N9 viruses are particularly associated with severe human disease [6,7,8]. Although direct-acting antivirals targeting viral proteins, including polymerase-targeting inhibitors such as baloxavir marboxil and onradivir, have been developed, the emergence of drug-resistant variants highlights the need for complementary therapeutic strategies [9]. Host-directed approaches that target cellular pathways exploited by IAV may offer broader-spectrum antiviral activity and potentially impose a higher genetic barrier to resistance [10]. Therefore, elucidating host mechanisms that regulate IAV replication is important for understanding virus–host interactions and for identifying new antiviral targets.

**Figure 1:**
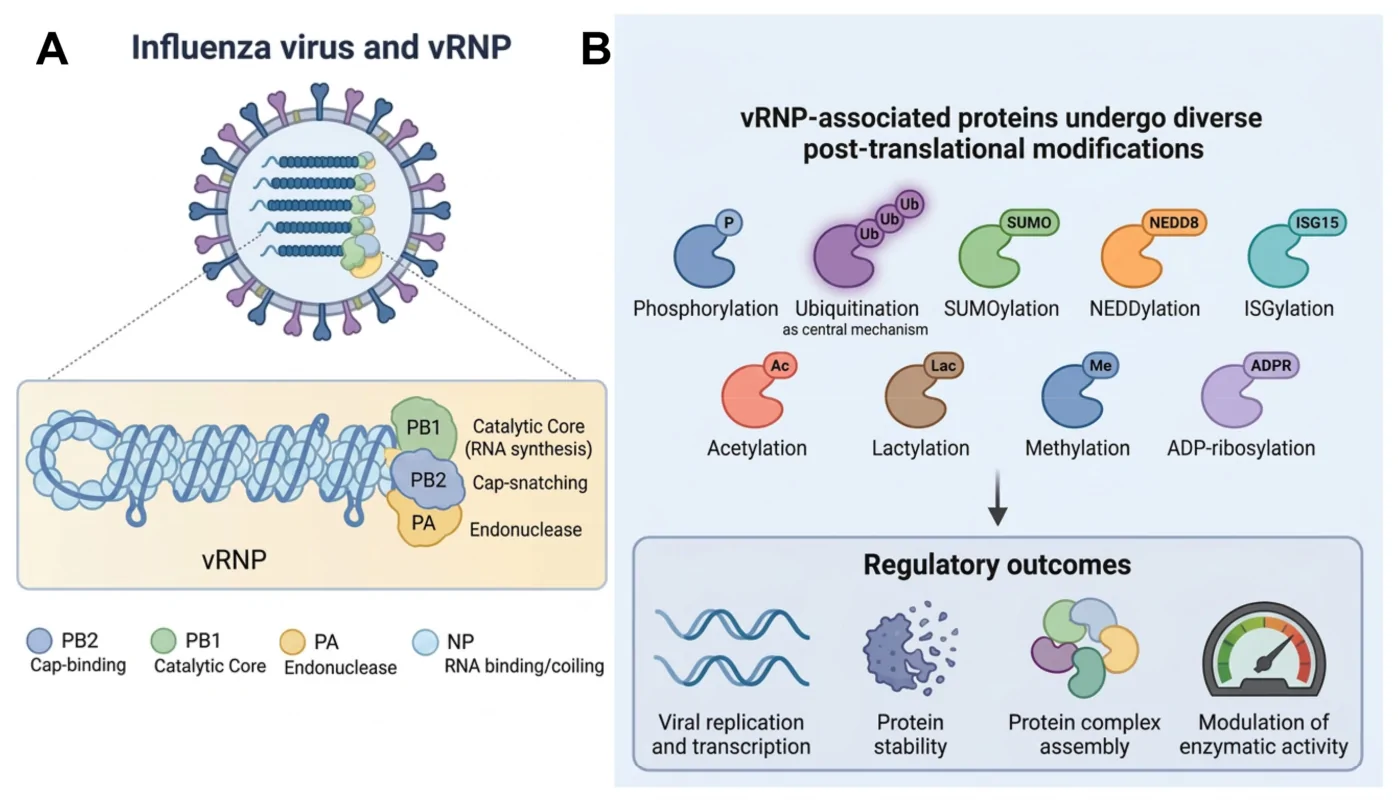
Post-translational Modifications of the Influenza vRNP. (**A**) The influenza virus vRNP consists of viral RNA (vRNA), nucleoprotein (NP), and the heterotrimeric RNA-dependent RNA polymerase (RdRp), which comprises the PB1, PB2, and PA subunits. (**B**) vRNP-associated proteins undergo a variety of post-translational modifications, including phosphorylation, acetylation, ubiquitination, SUMOylation, methylation, NEDDylation, ISGylation, lactylation, and ADP-ribosylation, thereby regulating viral replication, transcription, protein stability, subcellular localization, protein–protein interactions, and protein complex assembly. Panel B is a conceptual overview and does not imply that every listed modification has been demonstrated on every vRNP component or in a common infection model. This figure was generated with assistance from ChatGPT (GPT-5.5) and Nano Banana Pro (Gemini 3 Pro Image).

FluPol is essential for viral transcription and genome replication and represents an important determinant of host range and pathogenicity. FluPol is a heterotrimeric complex composed of PB1, PB2, and PA. PB1 forms the catalytic core and contains the conserved polymerase active site responsible for viral RNA synthesis. PB2 binds the 5′ cap structures of host pre-mRNAs, whereas PA contains an endonuclease domain that cleaves capped host RNA fragments, thereby generating primers for viral mRNA transcription through the cap-snatching mechanism [11,12]. In infected cells, one FluPol complex associates with each viral RNA segment together with multiple copies of NP to form vRNPs. Within vRNPs, NP encapsidates the viral RNA, while FluPol binds the conserved 5′ and 3′ termini of the viral genome to form the promoter structure required for transcription and replication. Accordingly, polymerase function is not determined solely by the intrinsic catalytic properties of PB1, PB2, and PA, but also depends on vRNP assembly, stability, nuclear trafficking, conformational dynamics, and functional integrity. These processes are extensively influenced by host factors and post-translational modifications (PTMs) [13,14,15] (Figure 1A).

Host-dependent regulation of FluPol is particularly relevant to viral host adaptation and interspecies transmission. Members of the acidic nuclear phosphoprotein 32 kDa (ANP32) family, especially ANP32A and ANP32B, are established host cofactors that support efficient IAV polymerase activity in mammalian cells [16]. Species-specific differences in ANP32 proteins contribute to the host restriction of avian influenza virus polymerases, whereas mammalian-adaptive substitutions in PB2, such as E627K, can enhance polymerase activity in mammalian cells, at least in part by improving compatibility with mammalian ANP32 cofactors [17]. In addition to host cofactors, influenza polymerase activity is regulated by diverse host-dependent PTMs, including phosphorylation [18,19], ubiquitination [20], SUMOylation [21,22], NEDDylation [23], ISGylation [24], acetylation [25,26,27,28], lactylation [29], methylation [30,31], and ADP-ribosylation [32]. These modifications can affect viral protein stability, subcellular localization, protein–protein interactions, RNA-binding capacity, immune evasion, and polymerase activity (Figure 1B). Thus, PTMs provide a dynamic and reversible regulatory layer through which host cells can restrict viral replication, while IAV may also exploit host modification systems to optimize its life cycle.

Among the diverse PTMs that regulate IAV polymerase function, ubiquitination deserves particular attention because of its structural diversity, reversibility, and capacity to mediate both degradative and non-degradative regulatory outcomes. Through monoubiquitination, multi-monoubiquitination, and polyubiquitin chains with distinct linkage types, including K48-, K63-, and K29-linked chains, ubiquitination can regulate protein stability, proteasomal degradation, complex assembly, conformational dynamics, intracellular trafficking, innate immune signaling, and protein–RNA interactions. Accumulating evidence indicates that ubiquitination modifies major components of the IAV vRNP complex, including PB1, PB2, PA, and NP, thereby influencing polymerase assembly, vRNP formation, viral RNA synthesis, and host antiviral responses. Importantly, the functional consequences of ubiquitination are context-dependent: ubiquitination may restrict IAV replication by promoting degradation or functional disruption of viral proteins, but it may also be exploited by the virus to stabilize polymerase components or enhance vRNP activity. Thus, the ubiquitin system constitutes a dynamic interface between host restriction mechanisms and viral replication strategies. Moreover, E3 ubiquitin ligases, DUBs, and ubiquitin-dependent degradation pathways are increasingly recognized as pharmacologically tractable targets, providing opportunities for host-directed or virus-targeted antiviral intervention [33,34]. Therefore, elucidating how ubiquitination regulates FluPol and NP is essential for understanding IAV replication, pathogenicity, and host adaptation, and may facilitate the development of new anti-influenza strategies.

## 2. Diverse Ubiquitin Modifications and Their Regulation of IAV Polymerase Activity

**Figure 2:**
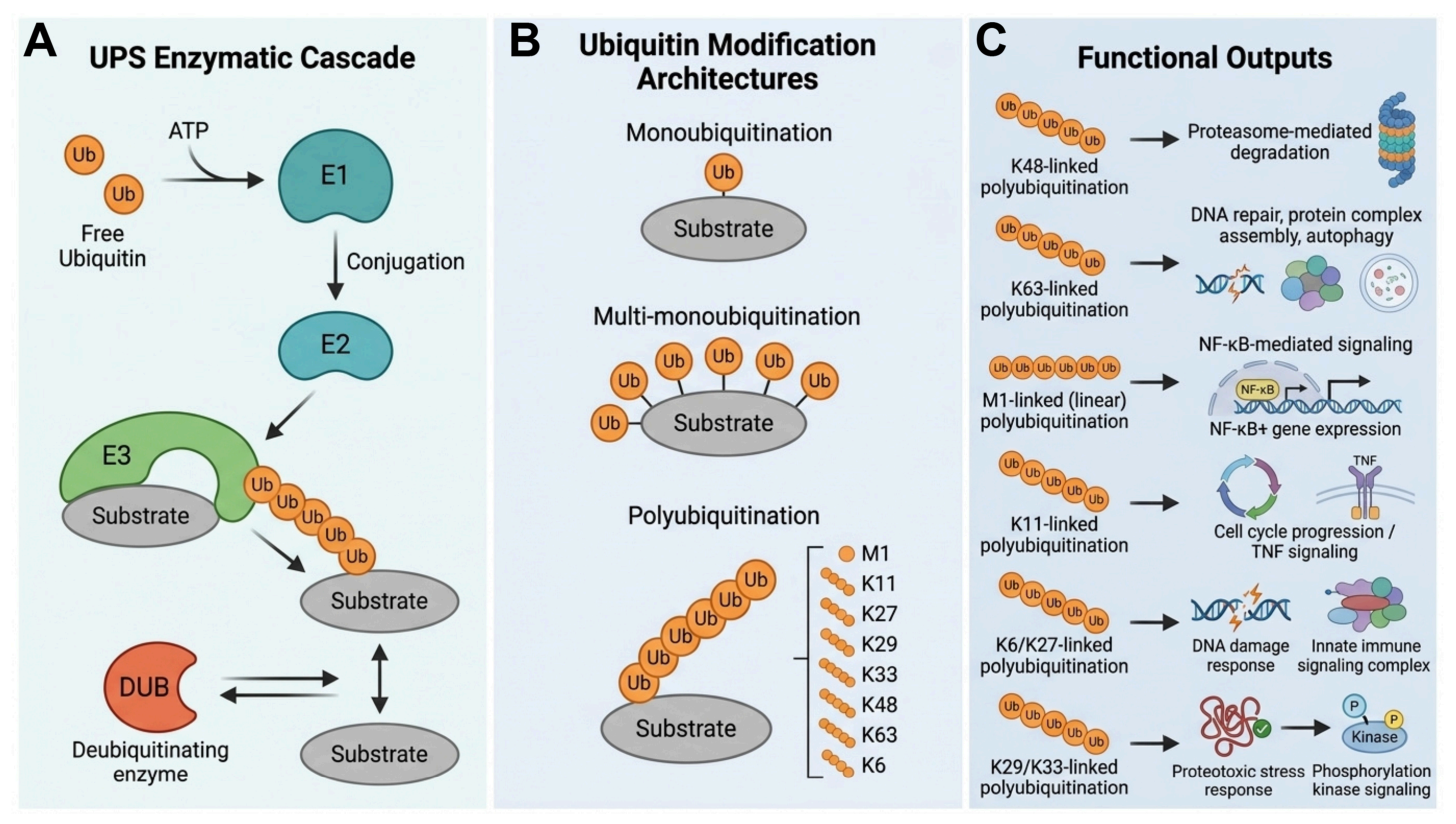
UPS and linkage-specific ubiquitin signaling. (**A**) The UPS enzymatic cascade: ubiquitin is activated by E1, transferred to E2, and ligated to substrate proteins by E3; this process can be reversed by DUBs. Arrows show transfer or regulation; orange “Ub” circles denote ubiquitin; colors distinguish UPS components and panel C outcomes. (**B**) Major ubiquitin architectures: monoubiquitination, multi-monoubiquitination, and polyubiquitination. (**C**) Linkage-specific functions: K48-linked chains direct substrates to the 26S proteasome for degradation, while M1, K6, K11, K27, K29, K33, and K63 linkages regulate distinct signaling pathways, including NF-κB signaling, DNA damage response, protein complex assembly, cell-cycle progression, innate immunity, proteotoxic stress, kinase signaling, and autophagy. This figure was generated with assistance from ChatGPT (GPT-5.5) and Nano Banana Pro (Gemini 3 Pro Image).

The ubiquitin–proteasome system (UPS) is a coordinated enzymatic network that regulates protein stability and cellular signaling. Ubiquitination is initiated by E1 ubiquitin-activating enzymes, transferred by E2 ubiquitin-conjugating enzymes, and catalyzed with substrate specificity primarily by E3 ubiquitin ligases, which attach ubiquitin predominantly to lysine residues on target proteins [35]. This modification is reversible and is removed or edited by DUBs, thereby remodeling or terminating ubiquitin-dependent signals [36,37,38]. The functional diversity of ubiquitination is determined by its structural complexity, including monoubiquitination, multi-monoubiquitination, and polyubiquitin chains linked through K6, K11, K27, K29, K33, K48, K63, or the N-terminal methionine M1 [39,40,41,42]. These distinct ubiquitin architectures are interpreted by ubiquitin-binding proteins and can encode different biological outcomes. K48-linked polyubiquitin chains are classically associated with 26S proteasome-mediated degradation, whereas many non-K48 linkages are involved in non-proteolytic processes, although their functions are often context-dependent. For example, M1-linked linear chains regulate NF-κB signaling; K6-linked chains have been implicated in DNA damage responses; K11-linked chains participate in cell-cycle regulation and TNF-related signaling; K27-linked chains contribute to innate immune signaling and have also been reported to mediate proteasome-dependent degradation in specific contexts [43,44]; K29-linked chains are associated with proteotoxic stress responses; K33-linked chains modulate AMPK-related kinase signaling; and K63-linked chains regulate DNA damage responses, protein complex assembly, signal transduction, and autophagy (Figure 2).

In the context of IAV infection, accumulating evidence indicates that ubiquitination regulates polymerase function not only by controlling viral protein turnover but also by directly modulating vRNP activity. Increased ubiquitin expression has been reported to enhance IAV polymerase activity in a dose-dependent manner, accompanied by increased accumulation of viral mRNA, complementary RNA (cRNA), and viral genomic RNA (vRNA) [45]. In addition, the major components of the vRNP complex, including PB1, PB2, PA, and NP, can undergo ubiquitination [20,45]. These observations support the concept that the UPS functions not merely as a degradative pathway but also as a dynamic and reversible signaling network during IAV replication. While K48-linked polyubiquitination commonly promotes proteasomal degradation of viral or host proteins, non-canonical ubiquitin modifications, such as K63-linked polyubiquitin chains and monoubiquitination, can regulate non-proteolytic processes, including protein stabilization, protein–protein interactions, vRNP assembly, intracellular trafficking, and modulation of enzymatic activity [39,40].

Global site-resolved analyses highlight the distinction between modification detection and mechanistic assignment. In A/WSN/1933-infected A549 cells, mass spectrometric analysis of immunopurified peptides bearing the K-epsilon-GG (di-glycyl) remnant identified 59 modified lysine residues in PB2 (22), PB1 (15), and PA (22). However, the di-glycyl remnant can derive from ubiquitin or ubiquitin-like modifiers, and most of these sites have not been individually assigned to a specific E3 ligase, ubiquitin linkage, or direct functional consequence. Accordingly, Table A2 distinguishes landscape-level candidate sites from those supported by direct functional, linkage-specific, or reverse-genetics evidence, and reports conservation only for sites that were directly assessed [20].

## 3. Molecular Mechanisms Underlying Ubiquitination-Mediated Regulation of IAV Polymerase Activity

IAV RNA synthesis is catalyzed by the viral RNA-dependent RNA polymerase, FluPol, which functions in association with viral RNA and nucleoprotein, NP, within viral ribonucleoprotein complexes, vRNPs. The activity of FluPol is not determined solely by the intrinsic catalytic properties of the PB1, PB2, and PA subunits. Instead, efficient viral transcription and replication require coordinated regulation of polymerase subunit stability, conformational dynamics, subcellular localization, vRNP assembly, and interactions with NP and other viral or host factors. Nonstructural viral proteins also contribute to this regulatory network. For example, NS1 and NS2 have been shown to coordinate dynamic viral RNA synthesis. Early during infection, NS1 promotes transcription through the RNA-binding residues R38 and K41, whereas late NS2 suppresses transcription and enhances replication, a regulatory pattern conserved in influenza A and B viruses. The N-terminal region of NS2, amino acids 1–20, mediates transcriptional inhibition, whereas the C-terminal residue I121 is required for replication promotion [46]. Within this multilayered regulatory framework, ubiquitination has emerged as a versatile post-translational modification that modulates IAV polymerase activity at multiple levels.

Ubiquitination regulates IAV polymerase function through several mechanistically distinct modes. First, degradative ubiquitination, particularly K48-linked polyubiquitination, reduces the abundance of polymerase subunits or NP, thereby limiting the formation of functional vRNPs and suppressing viral RNA synthesis. Second, non-degradative ubiquitination, including monoubiquitination and atypical polyubiquitin chains such as K63-linked chains, can modulate polymerase activity by altering protein–protein interactions, conformational transitions, and vRNP assembly. Third, DUBs can remove ubiquitin modifications from viral or host proteins, thereby stabilizing polymerase components or reversing regulatory ubiquitin signals. The following sections summarize how ubiquitin-dependent pathways regulate the abundance, assembly, and activity of the IAV polymerase machinery.

Critical interpretation of this literature requires separating the level of evidence for an antiviral phenotype from the level of evidence for the proposed molecular mechanism. In this review, a mechanism is termed well supported only when a host factor is perturbed in an infection setting and the viral phenotype is linked to viral-protein ubiquitination, stability, or a site-dependent effect. We describe a mechanism as context-bounded when infection or reverse-genetics data are available but the conclusion has been tested in one viral background, cell type, or host species. Events observed principally in ubiquitin-remnant proteomics, ectopic expression, minigenome assays, or structural modelling are referred to as emerging or candidate mechanisms. These labels refer to the evidence available for a proposed step, not to the biological importance of the host factor.

Notably, no individual FluPol or NP ubiquitination site discussed here can yet be regarded as independently validated across multiple research groups and multiple IAV lineages. Reported mechanisms therefore should not be extrapolated indiscriminately from one strain or host system to another. Where strain specificity has been directly examined, it is indicated below; otherwise, the relevant finding should be interpreted as a context-dependent observation rather than a universal feature of IAV infection.

### 3.1. Regulation of PB1 by E3 Ubiquitin Ligases

PB1 constitutes the catalytic core of FluPol and is essential for viral RNA synthesis. Therefore, ubiquitin-mediated regulation of PB1 stability or modification status can directly influence polymerase activity and viral replication. Several host E3 ubiquitin ligases restrict IAV replication by targeting PB1 for degradation. TRIM32 has been reported to promote K48-linked polyubiquitination and proteasomal degradation of PB1, thereby reducing the abundance of functional polymerase complexes and suppressing viral RNA synthesis [47]. Because PB1 provides the catalytic center of FluPol, TRIM32-mediated PB1 degradation directly compromises polymerase assembly and activity.

In addition to degradative ubiquitination, site-specific ubiquitination of PB1 may regulate polymerase function through non-degradative mechanisms. Functional analyses combined with structural modeling suggest that ubiquitination at PB1-K578, located within the thumb domain, may influence conformational transitions required for vRNA replication [20]. Ubiquitin-mediated charge neutralization at this site has been proposed to disrupt the interaction between PB1 and an unstructured loop in the PB2 N-terminus, a contact that may contribute to polymerase dimerization and efficient vRNA replication. Thus, PB1-K578 ubiquitination may act as an allosteric regulatory signal that modulates inter-subunit interactions and controls transitions between distinct functional states of the polymerase complex.

Notably, some E3 ligases can be exploited by IAV to support polymerase activity. TRIM31, an E3 ubiquitin ligase originally identified as a positive regulator of type I interferon responses through mitochondrial antiviral-signaling protein (MAVS), specifically interacts with PB1, PA, and HA proteins of various IAV subtypes. TRIM31 catalyzes K63-linked ubiquitination of these viral proteins, enhancing their stability and promoting polymerase or membrane fusion activity [48]. For PB1, TRIM31-mediated non-degradative ubiquitination may help maintain polymerase homeostasis and support efficient viral RNA synthesis. Meanwhile, competitive binding of PB1, PA, and HA to TRIM31 attenuates TRIM31–MAVS-mediated type I interferon (IFN-I) signaling, indicating that IAV can exploit TRIM31 both to enhance viral polymerase function and to dampen innate immune activation.

PB1 also participates in viral counteraction against host restriction factors. Zinc-finger antiviral protein long isoform (ZAPL) binds PA and PB2 through its C-terminal poly(ADP-ribose) polymerase (PARP) domain and promotes their proteasomal degradation via poly(ADP-ribosylation) and ubiquitination [32]. However, viral PB1 can bind near the PARP domain of ZAPL, induce the dissociation of PA and PB2 from ZAPL, and prevent their degradation. This mechanism preserves polymerase subunit stability and suggests that the PB1–ZAPL interface may represent a potential target for antiviral intervention.

### 3.2. Regulation of PB2 by E3 Ubiquitin Ligases

PB2 plays critical roles in cap binding, host adaptation, polymerase assembly, and vRNP function. Consequently, ubiquitin-mediated regulation of PB2 has profound effects on viral RNA transcription and replication. Several host E3 ligases restrict IAV replication by promoting PB2 degradation. TRIM35 targets PB2 for K48-linked ubiquitination and proteasomal degradation, thereby reducing PB2 availability and suppressing viral polymerase activity [49]. In addition to directly destabilizing PB2, TRIM35 may enhance retinoic acid-inducible gene I (RIG-I)-mediated innate immune signaling by promoting K63-linked polyubiquitination of tumor necrosis factor receptor-associated factor 3 (TRAF3), which further limits the ability of PB2 to antagonize antiviral responses. Thus, TRIM35 restricts IAV replication through both direct degradation of PB2 and indirect enhancement of host antiviral signaling.

PB2 stability is also regulated through crosstalk between ubiquitination and chaperone-mediated autophagy (CMA). LAMP-2A is the rate-limiting receptor for CMA and recognizes substrate proteins containing KFERQ-like motifs, including the QMRDV sequence at residues 602–606 of PB2 [50]. CAPN1, a calpain protease, cleaves lysosomal membrane-localized LAMP-2A and thereby limits CMA activity. TRIM45 mediates K48-linked polyubiquitination and proteasomal degradation of CAPN1, preventing CAPN1-dependent cleavage of LAMP-2A [51]. As a result, TRIM45 increases LAMP-2A abundance, enhances the PB2–LAMP-2A interaction, and promotes PB2 degradation through the CMA pathway [52]. This TRIM45–CAPN1–LAMP-2A axis decreases PB2 availability and restricts assembly and activity of the viral polymerase complex.

By contrast, certain E3 ligase-mediated ubiquitination events of PB2 are non-degradative and appear to support IAV replication. CRL4 E3 ligases associated with substrate-recognition factors such as DCAF11 and DCAF12L1 can attach K29-linked ubiquitin chains to specific lysine residues of PB2 [53,54]. DCAF12L1 targets PB2 at K482 and K752, whereas DCAF11 mainly modifies PB2 at K660/K663. These modifications promote viral replication, although they were reported to have limited effects on viral transcription or replication, suggesting that PB2 ubiquitination by these CRL4 complexes may regulate additional steps of the viral life cycle.

Host deubiquitinases also counterbalance PB2 ubiquitination. USP39 directly deubiquitinates PB2 at K660, preventing PB2 degradation and sustaining polymerase activity [55]. Independently of its catalytic activity, USP39 also promotes PB2–PB1 association, facilitating efficient vRNP complex formation in H5 avian influenza virus. These two functions—stabilization of PB2 and enhancement of PB2–PB1 interaction—cooperate to increase viral RNA synthesis and support efficient replication. In addition to E3 ligase-mediated pathways, ZAPL-mediated restriction also provides an example of host-driven PB2 destabilization. As described above, ZAPL promotes PB2 and PA degradation through mechanisms involving poly(ADP-ribosylation) and ubiquitination, whereas PB1 antagonizes this process to preserve polymerase subunit stability [32].

### 3.3. Regulation of PA by E3 Ubiquitin Ligases

PA provides the endonuclease activity required for cap-snatching and forms an essential structural interface with PB1. Therefore, ubiquitin-mediated control of PA abundance or stability directly affects the formation of transcriptionally competent polymerase complexes. DCAF7 acts as a substrate-recognition receptor within the cullin-RING ligase 4B (CRL4B)–DCAF7 E3 ligase complex and promotes K48-linked polyubiquitination of PA at K609, leading to PA proteasomal degradation [56]. Since PA is indispensable for cap-dependent transcription initiation and polymerase assembly, DCAF7-mediated PA degradation reduces the abundance of functional FluPol complexes and suppresses viral RNA synthesis. The viral NS1 protein has been reported to reduce DCAF7 abundance, suggesting that IAV may attenuate this antiviral restriction mechanism to preserve PA stability.

Similar to PB1, PA can also be positively regulated by proviral E3 ligases. TRIM31 interacts with PA and catalyzes K63-linked ubiquitination, which enhances PA stability and contributes to increased polymerase activity [48]. Meanwhile, PA competes with MAVS for TRIM31 binding, thereby weakening TRIM31-mediated IFN-I signaling. Thus, TRIM31 has dual effects during IAV infection: it can promote antiviral signaling under normal conditions, but IAV co-opts TRIM31 to stabilize viral proteins such as PA and enhance polymerase function while dampening innate immune activation. PA is also affected by the ZAPL-associated restriction pathway discussed above, supporting the importance of PA stability in maintaining FluPol assembly and activity [32].

Together, these studies suggest that PA is regulated by both antiviral and proviral ubiquitin pathways. Antiviral E3 ligases such as DCAF7 and ZAPL-associated ubiquitination pathways destabilize PA and restrict polymerase activity, whereas TRIM31-mediated K63-linked ubiquitination supports PA stability and viral RNA synthesis.

### 3.4. Regulation of NP and vRNP Activity by E3 Ubiquitin Ligases

NP is the major viral RNA-binding protein and a central component of the vRNP complex. It stabilizes viral RNA, organizes vRNP architecture, supports polymerase processivity, and mediates NP–RNA and NP–polymerase interactions. Therefore, ubiquitination-dependent regulation of NP abundance, modification status, or interaction networks can profoundly influence viral RNA synthesis and overall polymerase output. Compared with PB1, PB2, and PA, NP is regulated by a particularly diverse set of E3 ligases, and the outcome of NP ubiquitination is highly dependent on ubiquitin linkage type, modification site, chain length, and structural context.

Several E3 ubiquitin ligases restrict IAV replication by promoting NP degradation. TRIM22 mediates polyubiquitination of NP and promotes its proteasomal degradation, thereby reducing NP abundance and impairing vRNP formation [57]. TRIM41 recognizes NP through its SPRY domain and promotes NP ubiquitination, leading to inhibition of IAV replication [58]. TRIM14, although not a canonical really interesting new gene (RING)-type TRIM E3 ligase, has also been reported to interact with NP and promote NP destabilization, possibly through an indirect or adaptor-like mechanism [59]. In addition, Grail interacts with NP and targets it for degradation, thereby suppressing IAV replication. These NP-directed ubiquitination pathways reduce the availability of NP, destabilize vRNP templates, and ultimately inhibit polymerase-dependent viral RNA synthesis [60].

The TRIM4–ABTB1 axis further illustrates the dynamic balance between host restriction and viral counteraction. TRIM4 inhibits IAV replication through its E3 ubiquitin ligase activity by targeting both incoming vRNP-associated NP and newly synthesized NP for degradation [61]. Conversely, ABTB1 interacts with TRIM4 and promotes its proteasomal degradation, thereby preventing TRIM4-mediated NP destabilization. As a result, ABTB1 attenuates the antiviral effect of TRIM4, preserves NP abundance, and supports vRNP function.

In addition to degradative ubiquitination, some NP ubiquitination events positively regulate viral RNA synthesis. Monoubiquitination of NP at K184 has been proposed to enhance viral genome replication [62]. The E3 ubiquitin ligase CNOT4 targets several lysine residues of NP, including K184, K227, and K273, which are located within or near the RNA-binding groove of NP [62]. Mutation of these residues to arginine reduces viral RNA replication, indicating that CNOT4-mediated monoubiquitination enhances vRNP activity and promotes viral RNA synthesis. Notably, NP can also be acetylated at K184, the same residue implicated in monoubiquitination, suggesting potential crosstalk between ubiquitination and acetylation at this site [15]. Consistently, deubiquitination of NP at K184 by the host deubiquitinase USP11 decreases polymerase activity and reduces viral replication [63]. These findings indicate that, unlike polyubiquitination-induced degradation, selected site-specific monoubiquitination events can enhance NP function within the vRNP complex. The opposing activities of CNOT4-mediated ubiquitination and USP11-mediated deubiquitination may therefore fine-tune NP modification and maintain an optimal level of vRNP activity.

In contrast to CNOT4, the host E3 ubiquitin ligase Pirh2 inhibits NP function without necessarily promoting complete NP degradation. Pirh2 interacts with NP and mediates short-chain ubiquitination of NP at K351 [64]. This modification suppresses the NP–PB2 interaction and consequently inhibits vRNP formation. Notably, sequence analysis of 26,977 human IAV isolates collected between 2011 and 2020 showed that 99.18% encode lysine (K) at NP residue 351, whereas 95.3% of 9956 avian IAV isolates encode arginine (R) at this position. In line with this host-associated sequence difference, Pirh2 knockdown did not enhance the replication of avian-like H9N2-W1 or H9N2-C1 viruses, both of which naturally encode R351. These findings suggest that variation at NP residue 351 may influence sensitivity to Pirh2-mediated restriction and may be associated with evasion of the human antiviral response by avian-like influenza viruses.

TRIM25 also regulates vRNP function through mechanisms that are not solely dependent on canonical ubiquitin ligase activity. TRIM25 is well known to activate RIG-I by catalyzing K63-linked polyubiquitination of its N-terminal CARD domains, thereby promoting RIG-I oligomerization and MAVS-dependent antiviral signaling [65]. However, TRIM25 can also associate with nuclear vRNPs and inhibit the initiation of influenza viral RNA chain elongation independently of its ubiquitin ligase activity [66]. This finding suggests that TRIM proteins can restrict IAV polymerase function not only by promoting ubiquitin-dependent degradation, but also by directly interfering with vRNP-associated processes.

Collectively, NP ubiquitination is not a uniform antiviral or proviral signal. Degradative ubiquitination mediated by TRIM22, TRIM41, TRIM4, Grail, and possibly TRIM14 reduces NP abundance and destabilizes vRNPs, thereby restricting polymerase activity. In contrast, site-specific non-degradative ubiquitination mediated by CNOT4 or monoubiquitination at K184 can enhance NP function and stimulate viral RNA synthesis. Pirh2-mediated short-chain ubiquitination inhibits NP–PB2 interaction and vRNP formation, whereas TRIM25 can suppress vRNP activity through both ubiquitin-dependent immune activation and ubiquitin-independent interference with RNA chain elongation. Together, these findings identify NP as a key ubiquitin-regulated determinant of vRNP function, polymerase activity, and viral replication.

As summarized in Table A1, all listed host ubiquitin-system factors were evaluated in IAV infection settings and linked to polymerase/vRNP activity or a related viral phenotype; the table separately records minigenome/polymerase assays, viral-RNA measurements, host-factor perturbation, and mutant analyses. Applying this criterion, TRIM32-PB1, TRIM35-PB2, DCAF7-PA, USP11-NP, and Pirh2-NP mechanisms currently have comparatively strong infection-linked support because these studies combine host-factor perturbation during virus infection with defined viral phenotypes and mechanistic ubiquitination or deubiquitination readouts. TRIM35, DCAF7, USP11, and Pirh2 further connect the phenotype to a defined ubiquitin linkage or acceptor site and include genetic, recombinant-virus, animal, or strain-comparison evidence [49,56,63,64]. TRIM45-CAPN1-PB2 and USP39-PB2 are included with method labels constrained to the reported viral background and pathway: TRIM45 acts through the CAPN1-LAMP-2A-CMA axis rather than direct PB2 ubiquitination, whereas USP39 evidence is centered on H5 IAV PB2 stabilization and PB1-PB2 association [52,55]. In contrast, the functional significance of PB1-K578 is supported by ubiquitin-remnant mapping, mutational analysis, and polymerase or reverse-genetics assays, whereas the proposed PB1-PB2 allosteric effect remains model-supported and awaits direct biochemical testing [20]. For CRL4-dependent PB2 K29 ubiquitination and CNOT4-dependent NP monoubiquitination, infection and/or mutational data support a proviral role, but DCAF11/DCAF12L1-associated minireplicon and viral-RNA readouts did not show significant changes after host-factor depletion, and the generality of exact site-specific effects requires additional testing across viral lineages and experimental systems [53,62]. Validation methods for each host factor are summarized in Table A1.

**Figure 3:**
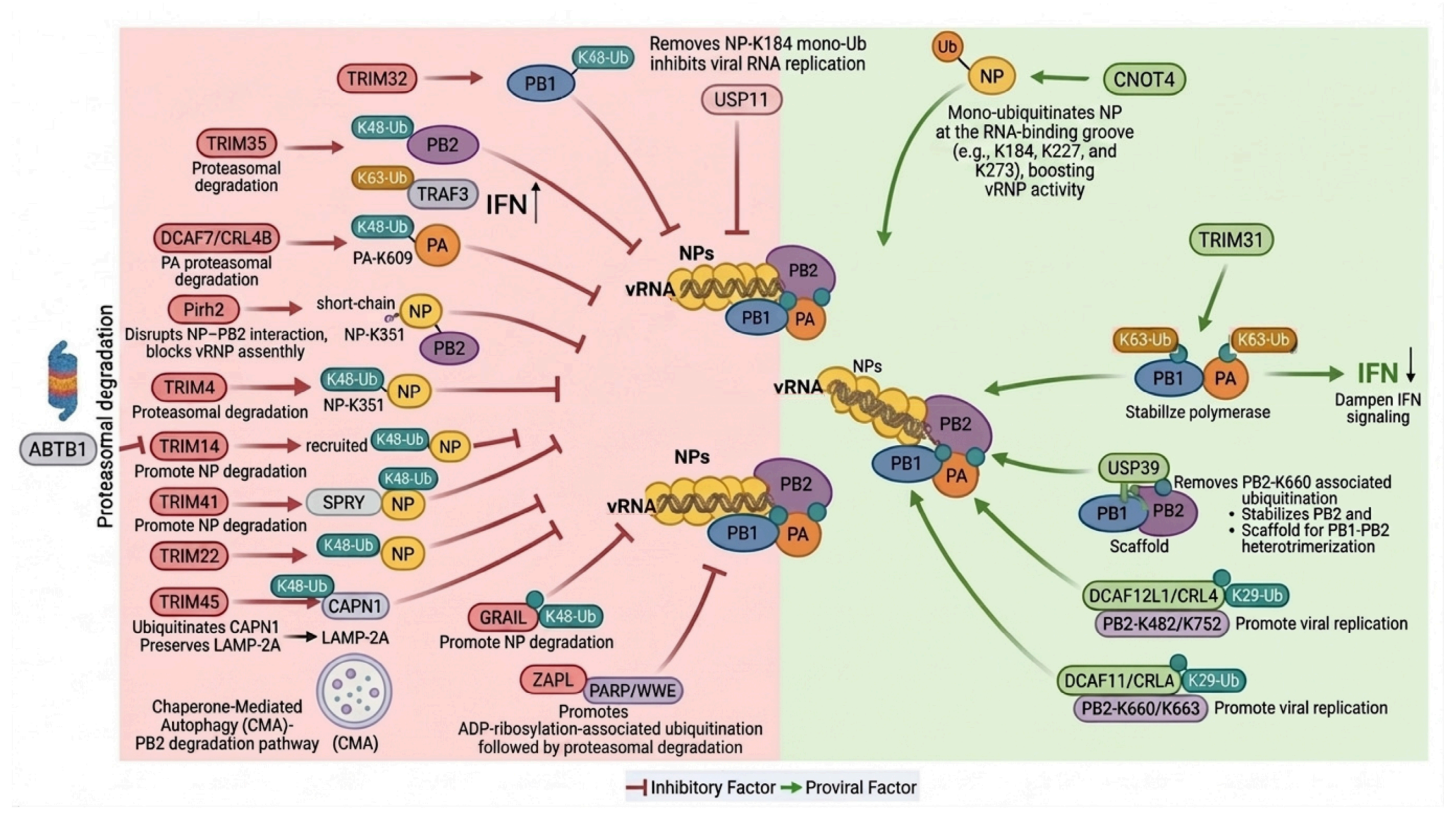
Dual regulation of IAV polymerase and NP by host ubiquitination and deubiquitination pathways. This schematic summarizes host ubiquitin-related regulators that modulate IAV polymerase complex activity, NP function, vRNP assembly, and viral RNA replication. The left red panel shows antiviral or inhibitory ubiquitin pathways that restrict IAV replication, mainly by promoting proteasomal degradation of polymerase subunits or NP, disrupting NP-PB2 interaction, impairing vRNP assembly, or enhancing interferon-associated antiviral responses. Representative factors include TRIM35-mediated PB2 degradation, DCAF7/CRL4B-mediated PA degradation, Pirh2-mediated short-chain ubiquitination of NP at K351, TRIM4-, TRIM14-, TRIM22-, TRIM41-, and GRAIL-associated NP degradation, and ZAPL-mediated ADP-ribosylation-associated ubiquitination and degradation of polymerase components. USP11 removes NP-K184 monoubiquitination and suppresses viral RNA replication. ABTB1 and TRIM45 regulate the chaperone-mediated autophagy pathway involving CAPN1 and LAMP-2A, thereby influencing PB2 degradation. The right green panel shows proviral ubiquitin pathways that enhance polymerase stability, vRNP activity, or viral replication. CNOT4 monoubiquitinates NP at residues within or near the RNA-binding groove, thereby promoting vRNP activity. TRIM31 mediates K63-linked ubiquitination of PB1 and PA, stabilizing the polymerase complex and dampening interferon signaling. USP39 removes PB2-K660-associated ubiquitination, stabilizes PB2, and acts as a scaffold for PB1-PB2 heterotrimerization. DCAF12L1/CRL4 and DCAF11/CRL4 promote K29-linked ubiquitination of PB2 at K482/K752 and K660/K663, respectively, thereby enhancing viral replication. Red blunt-ended lines indicate inhibitory effects, whereas green arrows indicate proviral effects. Symbols denote the labeled host factors, viral proteins, and complexes; red and green backgrounds group inhibitory and proviral pathways, respectively. Ub, ubiquitin; IFN, interferon; vRNA, viral RNA; NP, nucleoprotein; PB1/PB2/PA, IAV polymerase subunits; vRNP, viral ribonucleoprotein complex; CMA, chaperone-mediated autophagy. The arrows and blunt-ended lines summarize regulatory relationships reported in the cited studies; their integrated arrangement is a conceptual synthesis and does not imply that every linkage, substrate effect, or downstream consequence has been validated in the same viral strain, cell type, or infection model. This figure was generated with assistance from ChatGPT (GPT-5.5) and Nano Banana Pro (Gemini 3 Pro Image).

Overall, the activity of the IAV polymerase complex is shaped by a dynamic balance between host restriction and viral counteraction (Figure 3). For PB1, TRIM32-mediated degradation suppresses polymerase activity, whereas TRIM31-mediated K63-linked ubiquitination and PB1-dependent antagonism of ZAPL support polymerase stability and function. For PB2, TRIM35 and the TRIM45–CMA axis promote PB2 degradation and restrict replication, whereas CRL4–DCAF11/DCAF12L1-mediated non-degradative ubiquitination and USP39-mediated deubiquitination help maintain PB2 function. For PA, DCAF7 and ZAPL-associated pathways destabilize PA, while TRIM31 enhances PA stability and polymerase activity. For NP, multiple E3 ligases either degrade NP and impair vRNP formation or introduce site-specific non-degradative modifications that enhance vRNP activity. Therefore, ubiquitin-related regulation of PB1, PB2, PA, and NP constitutes a central mechanism controlling IAV polymerase activity and represents a promising target space for host-directed antiviral strategies.

This dual-panel organization is intended to emphasize bidirectional and context-dependent regulation, not to define ubiquitination as predominantly restrictive or predominantly pro-viral across all infection settings.

## 4. Host Adaptation and Species-Specific Ubiquitin Networks

A useful framework for host adaptation is to distinguish viral sequence determinants from host-network determinants, including the conservation and strain specificity of virus–host interactions. A previous computational and RNAi-screening study identified a human protein network regulating H5N1 polymerase activity in comparison with a mammalian-adapted H1N1 polymerase; among 31 tested host proteins, 18 (58%) were required for polymerase function in both viral contexts, whereas factors such as DDX17 showed PB2 genotype- and host–cell-dependent effects [14]. Although DDX17 is not an E3 ubiquitin ligase, this example illustrates that some polymerase-associated host interactions are conserved across viral contexts, whereas others are strain- or host-specific. On the viral side, substitutions can remove an acceptor lysine, alter a local E3/DUB recognition surface, or change the structural accessibility of a modified residue. On the host side, species differences in the abundance, inducibility, localization, substrate preference, or cofactor dependence of E3 ligases and DUBs can alter the same viral protein modification. This framework is conceptually analogous to the species-specific ANP32 requirement of the influenza polymerase, but it should not be assumed that a given ubiquitin regulator creates a species barrier until this has been tested in matched infection systems [14,16,17].

NP residue 351 illustrates a defined viral determinant within this framework. Pirh2-mediated short-chain ubiquitination at NP-K351 impairs NP-PB2 interaction and restricts replication in the tested setting, whereas an arginine is strongly enriched at this position among the avian isolates analyzed and the corresponding avian-like viruses were insensitive to Pirh2 depletion [64]. This observation supports a residue- and strain-dependent escape model, but it does not establish that all zoonotic viruses evade Pirh2 or that Pirh2 is a general human species barrier. In contrast, PB1-K578 is highly conserved across human, avian, and swine sequences in the available analysis, suggesting that this site is unlikely to explain broad host-range shifts through loss of the acceptor lysine itself [20].

Four questions should guide future comparisons: whether zoonotic strains alter E3/DUB recognition motifs; whether modified lysines show lineage-specific selection after accounting for polymerase functional constraints; whether the same virus acquires different ubiquitin/ubiquitin-like modifier (UBL) profiles in avian, swine, and human cells; and whether host-specific ubiquitin networks interact with established polymerase cofactors such as ANP32. Addressing these questions will require matched infections with defined viral backbones, quantitative ubiquitinomics, reverse-genetics tests of candidate residues, and measurements of host E3/DUB expression and activity. Until such comparisons are available, species-specific ubiquitin profiles and positive selection of ubiquitination sites should be treated as important hypotheses rather than established mechanisms.

## 5. PTM Crosstalk in the Control of FluPol and NP

Ubiquitination should be interpreted within a wider PTM network rather than as a stand-alone control layer. In principle, acetylation, SUMOylation, NEDDylation, and ubiquitination can compete for lysine residues, whereas phosphorylation, methylation, lactylation, and ADP-ribosylation can alter local conformations, protein interactions, or enzyme recruitment. However, the current literature generally documents individual modifications in separate experimental systems; shared residues or pathways should not be equated with direct PTM crosstalk without temporal, site-occupancy, or enzyme-dependence evidence [13,15,18,19,21,22,23,24,25,26,27,28,29,30,31].

NP-K184 provides the clearest potential convergence point in the present literature. This residue has been reported to undergo acetylation and CNOT4-associated monoubiquitination, while USP11-mediated removal of NP-K184 monoubiquitination suppresses polymerase activity [25,26,62,63]. These observations are compatible with competition or sequential regulation at K184, but the cited studies did not measure dual occupancy of acetylation and ubiquitination in infected cells. Similarly, PIAS1-mediated SUMOylation of PB2 has been proposed to enable ubiquitination-dependent PB2 degradation, whereas PB2 NEDDylation and CRL4-mediated K29 ubiquitination have distinct reported effects on stability or replication [22,23,53,54]. These examples should therefore be described as candidate crosstalk models unless the order of modification and the relevant enzymatic dependencies have been directly established.

The available evidence supports three testable models: direct competition at a shared lysine, modification cascades in which one PTM recruits a ubiquitin-system regulator, and parallel control at distinct residues. For example, PB1 K612 SUMOylation enhances viral RNA binding, whereas PB1 K578 ubiquitination regulates dimerization and vRNA replication; PA Y393 phosphorylation and N-terminal PA acetylation have opposing reported effects on polymerase-related functions; and ZAPL couples ADP-ribosylation with ubiquitin-associated degradation of PA and PB2 [19,21,28,32]. Together, these observations identify hypotheses for a PTM network, not an established unified PTM code. Resolving the network will require infection-based, occupancy-resolved proteomics combined with site-specific viral mutants and perturbation of the relevant E3 ligases, DUBs, and other PTM enzymes.

## 6. Cross-Cutting Mechanistic and Evolutionary Questions

The temporal and functional hierarchy among PTMs remains an important unresolved issue in FluPol regulation. Current evidence is insufficient to establish a fixed infection-stage order among ubiquitination, SUMOylation, acetylation, and other lysine-directed modifications on PB1 or the broader vRNP. Rather, these modifications are more likely to operate as a context-dependent regulatory network shaped by infection timing, local enzyme abundance, vRNP localization, substrate accessibility, and competition at the same or neighboring lysines. Enzyme stoichiometry and spatial localization may both influence this regulatory balance, but neither has been established as the dominant determinant during authentic IAV infection [20,21,25,28,32].

Non-proteasomal ubiquitination should therefore be treated as a core component of the model rather than as an exception to degradation. Reported ubiquitination at PB1-K578, CRL4-dependent K29-linked ubiquitination of PB2, TRIM31-mediated K63-linked ubiquitination of PB1/PA, and CNOT4-dependent NP monoubiquitination illustrate how ubiquitin can regulate polymerase conformation, inter-subunit assembly, protein stability, NP-RNA interaction, or vRNP activity without necessarily targeting the substrate for proteasomal degradation. However, direct structural evidence for specific ubiquitin-driven allosteric mechanisms, such as exposure of catalytic elements or masking of RNA-binding surfaces, remains limited, and allosteric models should be described as experimentally testable hypotheses unless supported by infection-linked biochemical or structural validation [20,48,53,54,62].

Although ubiquitinated FluPol or NP could theoretically be incorporated into assembling vRNPs and retained within mature virions, this possibility remains unproven and should be interpreted cautiously. Each ubiquitin moiety adds approximately 8.5 kDa to its substrate; therefore, extensive polyubiquitination, particularly long ubiquitin chains, may impose steric or structural constraints on vRNP organization and virion assembly. For example, TRIM31-mediated K63-linked polyubiquitination of PA and PB1 stabilizes these polymerase subunits and supports the view that non-degradative ubiquitin chains can enhance polymerase function [48]. Nevertheless, whether polymerase subunits carrying extensive polyubiquitin chains can be efficiently incorporated into virions is unknown. By contrast, monoubiquitination may be more structurally compatible with virion-associated vRNP components. CNOT4-mediated monoubiquitination of NP at residues within or near the RNA-binding groove, including K184, K227, and K273, enhances vRNP activity [62]. However, whether this modification is transient during intracellular vRNP assembly or persists after packaging into mature virions has not been experimentally determined. The existence of ubiquitinated FluPol or NP within mature virions therefore remains unresolved. Current evidence derives primarily from infected-cell lysates, reconstituted polymerase systems, minigenome assays, and ubiquitin-remnant proteomics rather than from density-purified virions [20,45].

The apparent selectivity of E3 ligase recruitment is unlikely to be governed by a single virus-encoded selection mechanism. Instead, substrate selection may reflect infection-stage expression, nuclear-cytoplasmic partitioning, adaptor availability, exposure of degrons or lysine patches, vRNP conformation, and competition between viral substrates and innate-immune substrates. This helps explain why the same ubiquitin-system factor can have different outputs. In different experimental contexts, TRIM31 has been reported to support ubiquitination of viral PB1/PA/HA while also intersecting with MAVS signaling, and TRIM25 can activate RIG-I yet has also been reported to restrict vRNP function through a ligase-independent mechanism. These examples support a substrate-switching model that requires endogenous, infection-stage-resolved validation rather than overexpression alone [48,65,66].

DUB-mediated regulation should likewise be interpreted in both viral-protein and innate-immune contexts. Removing ubiquitin from FluPol or NP may stabilize a proviral complex, but the same DUB or pathway-level perturbation could also affect K63-linked ubiquitin signaling required for RIG-I/MAVS pathway activation. Consequently, DUB-targeted antiviral strategies must distinguish direct effects on viral polymerase substrates from indirect suppression of antiviral sensing and should be evaluated with pathway-selective readouts rather than polymerase activity alone [55,62,65].

Host adaptation and lysine conservation impose further constraints on any simple restriction-only model. Although ubiquitin and many ubiquitin-system enzymes are conserved across mammals, viral polymerase ubiquitination can differ with viral lineage, host–cell environment, cofactor compatibility, and local sequence context. Lysine-to-arginine or lysine-loss changes may reduce sensitivity to a particular E3 ligase, but they can also compromise RNA binding, polymerase assembly, nuclear trafficking, structural stability, or interactions with cellular cofactors. Thus, observed lower ubiquitination of avian or swine FluPol in human cells should be considered a potential host-adaptation signal or compatibility phenotype, not direct proof of either a zoonotic barrier or a universally beneficial escape mechanism [17,20,64].

Finally, many E3-ligase mechanisms still need physiological calibration. Findings obtained by overexpressing viral proteins or ubiquitin-system enzymes in HEK293 or other reconstituted systems are valuable for mechanism discovery, but they can exaggerate weak or non-physiological interactions. Confidence is strongest when overexpression, minigenome, and proteomics data are followed by endogenous host-factor perturbation during authentic infection, recombinant viruses carrying site-specific mutants, and validation in primary human airway epithelial cultures or other respiratory-relevant models. This evidence boundary supports a balanced conceptual framework. In this framework, FluPol ubiquitination is neither predominantly restrictive nor predominantly pro-viral across all settings but acts as a context- and stage-dependent regulatory layer [20,47,49,53,56,62,64].

## 7. Therapeutic Potential and Constraints of Targeting Ubiquitin Pathways in IAV

Ubiquitin pathways offer several potential points of intervention in IAV replication because host E3 ligases and DUBs regulate the abundance and assembly of PB1, PB2, PA, and NP. Inhibiting a proviral DUB or an E3 ligase that installs a replication-supporting ubiquitin signal could reduce viral RNA synthesis. This approach is not yet therapeutically established. Because the same enzymes act on many cellular substrates, candidate inhibitors must separate antiviral activity from disruption of essential host functions.

Targeted degradation is one possible application, but current evidence in influenza remains indirect. Endogenous E3 ligases can reduce the abundance of PB1, PB2, PA, or NP [47,49,56,57], yet this does not show that a drug-like PROTAC can recruit those ligases to viral proteins in infected airway cells. A viable degrader would require a ligand for an accessible viral epitope and an E3 ligase that is active in the relevant cell type. It would also need to remove the intended viral protein without broadly perturbing host ubiquitin signaling. CRBN and VHL are plausible recruitment systems, but their suitability for influenza targets has not been demonstrated [67].

Computational tools may help prioritize ligands and linker geometries after suitable target and E3 ligase pairs have been defined experimentally. DeepDegradome illustrates this design role, but it does not provide evidence that an influenza-specific degrader will function in infected cells [68]. A recent nanobody-based degrader has provided proof of principle for engineered anti-influenza protein degradation [69]. That result should not be treated as evidence for small-molecule PROTACs against FluPol or NP, because the two modalities differ in intracellular delivery and target engagement. The immediate priorities are direct target engagement, selective depletion of the viral protein, antiviral activity in respiratory models, and an acceptable effect on host–cell viability.

## 8. Conclusions and Perspectives

Ubiquitination has emerged as an important regulatory mechanism controlling IAV vRNP function. Rather than acting solely as a canonical degradation signal, ubiquitination regulates viral RNA synthesis through multiple mechanisms, including modulating the stability of PB1, PB2, PA, and NP, reshaping viral protein–protein and protein–RNA interactions, altering polymerase conformation, and controlling vRNP assembly and activity. Depending on substrate identity, ubiquitin linkage type, modification site, chain length, the responsible E3 ligases or DUBs, and cell type- and infection stage-specific factors, ubiquitination can either restrict IAV replication as part of host antiviral defense or be exploited by the virus to promote efficient RNA synthesis. Thus, ubiquitination of FluPol and NP represents a dynamic regulatory network linking viral transcription, genome replication, vRNP assembly, host restriction, and viral adaptation. For this reason, the conceptual framework should remain balanced: ubiquitination can operate as host restriction, viral exploitation, or reciprocal host–virus regulation depending on substrate, linkage, site, enzyme, cell type, host species, and infection stage.

Despite recent progress, the global ubiquitination landscape of FluPol and NP remains incompletely understood. Mass spectrometry-based studies have identified numerous potential ubiquitination sites in PB1, PB2, PA, and NP, but only a limited number have been functionally characterized. For many modifications, the responsible E3 ubiquitin ligases, deubiquitinases, adaptor proteins, substrate-recognition mechanisms, and ubiquitin-chain architectures remain unclear. Current studies have mainly focused on K48-, K63-, and K29-linked ubiquitin chains, whereas the roles of K6-, K11-, K27-, K33-, and M1-linked ubiquitin chains, as well as mixed and branched ubiquitin architectures, remain largely unexplored in the context of FluPol and NP regulation. In addition, many findings are derived from overexpression systems, minigenome assays, or selected viral strains and therefore require validation in authentic infection models, recombinant viruses carrying site-specific mutations, physiologically relevant cell systems, and diverse IAV subtypes. Because FluPol activity is shaped by viral strain, host species, and cell type, ubiquitination-dependent mechanisms should be compared across avian, swine, and human IAV strains. Such studies will be necessary to determine how specific ubiquitination events contribute to viral replication, pathogenicity, host adaptation and restriction.

Moreover, for most ubiquitination sites identified on FluPol and NP, the corresponding DUBs remain unknown or poorly characterized. Systematic mapping of DUB–substrate pairs using clustered regularly interspaced short palindromic repeats (CRISPR)-based DUB knockout/activation libraries, proximity labeling, ubiquitin remnant profiling, or activity-based DUB probes will be essential for reconstructing the ubiquitin-dependent regulatory network governing FluPol and NP functions. Whether ubiquitination and other PTMs occur sequentially, competitively, or cooperatively on the same viral protein remains largely unresolved. Future studies should define how ubiquitination interacts with phosphorylation, acetylation, SUMOylation, ISGylation, and ADP-ribosylation to fine-tune FluPol and NP functions. Such PTM crosstalk may be particularly important at conserved lysine residues that act as molecular switches controlling polymerase activity, vRNP assembly, nuclear trafficking, host-factor interactions, and host adaptation.

From an evolutionary perspective, it remains unknown whether circulating seasonal IAV strains or newly emerging zoonotic subtypes (H5N1 clade 2.3.4.4b) have acquired mutations that alter their susceptibility to host E3 ligases or DUBs. Comparative pan-proteomics across viral isolates may reveal adaptive signatures that circumvent ubiquitin-dependent restriction. Integrating quantitative ubiquitinomics, global proteomics, reverse genetics, structural biology, CRISPR-based screening, and physiologically relevant infection models will be essential for constructing a comprehensive regulatory map of FluPol and NP. These approaches may also clarify whether ubiquitination contributes to IAV host adaptation, pathogenicity, and interspecies transmission.

From a therapeutic perspective, ubiquitin-dependent regulation of FluPol and NP provides new opportunities for anti-IAV drug development. Recent advances in targeting the ubiquitin system, including E3 ligase-directed pharmacology, DUB inhibitors, PROTACs, and molecular glues, have expanded the therapeutic potential of ubiquitin-dependent pathways [33,34,67]. Inhibiting proviral DUBs, blocking key E3–viral substrate interactions, or redirecting the UPS to selectively degrade essential viral proteins through PROTACs, molecular glues, or nanobody-based degraders may offer alternative antiviral strategies. However, selectivity, host toxicity, pathway redundancy, antiviral breadth, resistance potential, and degradation specificity remain major barriers to antiviral translation. Identification of conserved and functionally indispensable viral epitopes, together with rigorous validation in relevant infection models, will be critical for translating mechanistic insights into therapeutic applications.

## Data Availability

No new data were created or analyzed in this study. Data sharing is not applicable to this article.

## Abbreviations

The following abbreviations are used in this manuscript:

IAV	Influenza A virus
RdRp	RNA-dependent RNA polymerase
FluPol	Influenza virus RNA-dependent RNA polymerase
NP	Nucleoprotein
DUBs	Deubiquitinases
PTMs	Post-translational modifications
ANP32	Acidic nuclear phosphoprotein 32 kDa
UPS	Ubiquitin–proteasome system
cRNA	Complementary RNA
vRNA	Viral genomic RNA
IFN-I	Type I interferon
ZAPL	Zinc-finger antiviral protein long isoform
CMA	Chaperone-mediated autophagy
PROTAC	Proteolysis-targeting chimera
PB1	Polymerase basic protein 1
PB2	Polymerase basic protein 2
PA	Polymerase acidic protein
vRNP	Viral ribonucleoprotein
Ub	Ubiquitin
UBL	Ubiquitin-like modifier
E3	Ubiquitin ligase enzyme
RING	Really interesting new gene
CRL4	Cullin-RING ligase 4
MAVS	Mitochondrial antiviral-signaling protein
RIG-I	Retinoic acid-inducible gene I
TRAF3	TNF receptor-associated factor 3
K	Lysine
R	Arginine
CRISPR	Clustered regularly interspaced short palindromic repeats

## Appendix A

**Table A1:** Summary of host factors involved in ubiquitination-mediated regulation of IAV polymerase activity.

Host Factor	Category	Viral Target	Ub-Linkage Type	Functional Impact on Polymerase	Mechanism	Validation Methods
TRIM32 [47]	E3 Ligase	PB1	K48-linked (degradative ubiquitination)	Inhibitory	Promotes proteasomal degradation of PB1 subunits	Minigenome/polymerase luciferase assay; authentic IAV infection; TRIM32 overexpression, siRNA knockdown and genetic knockout/rescue; catalytic RING and ubiquitin-chain mutant analysis.
TRIM35 [49]	E3 Ligase	PB2	K48-linked PB2 ubiquitination (degradative); K63-linked TRAF3 ubiquitination (non-degradative)	Inhibitory	Mediates PB2 degradation and activates RIG-I signaling via TRAF3	Minigenome/polymerase luciferase assay; authentic IAV infection and genetic-deficiency validation; TRIM35 overexpression/siRNA perturbation; TRIM35 RING/catalytic, ubiquitin-linkage and PB2-K736 mutant analysis.
DCAF7/CRL4B [56]	E3 Ligase	PA	K48-linked (degradative ubiquitination)	Inhibitory	Promotes polyubiquitination at PA-K609, leading to subunit degradation	Minigenome/polymerase luciferase assay; authentic IAV infection and in vivo validation; vRNA/cRNA/mRNA quantification; DCAF7 overexpression, siRNA and knockout; ubiquitin-linkage and PA-K609/site-mutant analysis.
Pirh2 [64]	E3 Ligase	NP	Short-chain ubiquitination (non-degradative ubiquitination)	Inhibitory	Mediates short-chain ubiquitination of NP at lysine 351; impairs NP–PB2 interaction and RNP assembly	Minigenome/polymerase luciferase assay; authentic IAV infection; infected-cell viral PA mRNA RT-qPCR; Pirh2 overexpression, siRNA and knockout; Pirh2 ΔRING/dominant-negative and NP-K351 mutant/recombinant-virus analysis.
TRIM4 [61]	E3 Ligase	NP	K48-linked (degradative ubiquitination)	Inhibitory	TRIM4 degrades NP and inhibits replication; ABTB1 counteracts this by promoting TRIM4 degradation.	Authentic IAV infection; ABTB1/TRIM4 overexpression, siRNA and knockout; ubiquitin-linkage and TRIM4 isoform/domain analysis; NP ubiquitination/degradation and vRNP nuclear-import readouts.
TRIM14 [59]	Non canonical RING-type TRIM E3 ligase	NP	K48-linked (degradative ubiquitination)	Inhibitory	TRIM14 lacks a canonical RING domain; describe as indirect or recruited-E3 NP degradation rather than a conventional E3 mechanism.	vRNP minigenome/reconstitution assay; IAV infection/reporter-virus validation; reconstituted-system vRNA/mRNA RT-qPCR; TRIM14 overexpression and knockout; TRIM14 domain-mutant analysis.
TRIM41 [58]	E3 Ligase	NP	K48-linked (degradative ubiquitination)	Inhibitory	TRIM41 binds NP via its SPRY domain and promotes NP ubiquitination, triggering NP degradation	Authentic IAV infection; TRIM41 overexpression, RNAi knockdown and CRISPR knockout; TRIM41 ΔRING/SPRY-domain mutant analysis; NP ubiquitination/degradation assays.
TRIM22 [57]	E3 Ligase	NP	K48-linked (degradative ubiquitination)	Inhibitory	Triggers NP degradation	Authentic IAV infection; TRIM22 overexpression and shRNA depletion; TRIM22 ΔRING mutant analysis; NP ubiquitination/degradation assays.
TRIM45 [52]	E3 Ligase	CAPN1, indirect PB2 restriction	K48-linked (degradative ubiquitination)	Inhibitory	Targets CAPN1 for degradation, preserves LAMP-2A, and facilitates CMA-dependent PB2 degradation	Authentic IAV infection and in vivo validation; TRIM45 overexpression, siRNA and knockout/rescue; TRIM45 RING, PB2 QMRDV/Q602A, CAPN1-K654 and ubiquitin-linkage mutant analysis.
GRAIL [60]	E3 Ligase	NP	K48-linked (degradative ubiquitination)	Inhibitory	GRAIL interacts with NP, increases NP ubiquitination, reduces NP abundance	Authentic IAV infection and in vivo validation; GRAIL overexpression, shRNA knockdown and genetic knockout; NP ubiquitination/degradation assays.
ZAPL [32]	Ubiquitin-dependent non-E3 regulator	PA/PB2	ADP-ribosylation-associated Ub (degradative ubiquitination)	Inhibitory	ZAPL binds PA/PB2 through the PARP/WWE region and promotes ubiquitination followed by proteasomal degradation	Virus infection; ZAPL overexpression and siRNA knockdown; ZAPL C88R/domain-mutant analysis; plasmid-derived PA mRNA RT-qPCR (indirect; not an authentic-infection viral RNA readout).
CNOT4 [62]	E3 Ligase	NP	Mono-Ub (non-degradative ubiquitination)	Proviral	Targets RNA-binding groove of NP (K184, K227, K273); boosts vRNP activity	Minigenome/polymerase luciferase assay; authentic IAV infection; infected-cell viral NP RNA RT-qPCR; CNOT4 overexpression and shRNA depletion/rescue; NP-K184/K227/K273 site-mutant analysis.
TRIM31 [48]	E3 Ligase	PB1, PA	K63-linked (non-degradative ubiquitination)	Proviral	Enhances PA and PB1 stability via K63-linked chains and dampens IFN signaling	Minigenome/polymerase luciferase assay; authentic IAV infection; TRIM31 overexpression and knockdown/rescue; RING/catalytic and ubiquitin-linkage mutant analysis.
USP11 [63]	DUB	NP	K184 mono-Ub removal (DUB-mediated regulation, reversible ubiquitination regulation)	Inhibitory	Inhibits influenza A virus RNA replication by K184 mono-Ub removal	Minigenome/polymerase luciferase assay; authentic IAV infection; primer-extension/qRT-PCR analysis of vRNA/cRNA/mRNA; USP11 overexpression and knockdown; catalytic USP11 and NP-K184 mutant/recombinant-virus analysis.
USP39 [55]	DUB	PB2	K660 removal (DUB-mediated regulation)	Proviral	Stabilizes H5N1 PB2 at K660 and scaffolds PB1–PB2 heterotrimerization	Minigenome/polymerase reporter assay; authentic H5 IAV infection and in vivo validation; infected-cell M1 mRNA/vRNA quantification; USP39 overexpression and knockout; catalytic USP39 and PB2-K660/site/domain mutant analysis.
DCAF12L1/CRL4 [53]	Cullin 4-Based E3 ligase	PB2	K29-linked (non-degradative ubiquitination)	Proviral	Contributes to CRL4-mediated PB2 ubiquitination, mainly targeting PB2 K482 and K752; promotes viral replication, with the downstream step remaining unresolved	Minigenome/polymerase luciferase assay; authentic IAV infection; strand-specific vRNA/mRNA RT-qPCR; CRL4-factor overexpression and siRNA depletion; ubiquitin-linkage and PB2 lysine-site mutant/recombinant-virus analysis; polymerase and viral-RNA readouts showed no significant effect following host-factor depletion.
DCAF11/CRL4 [53]	Cullin 4-Based E3 ligase	PB2	K29-linked (non-degradative ubiquitination)	Proviral	Contributes to CRL4-mediated PB2 ubiquitination, mainly targeting PB2 K660/K663; promotes viral replication, with the downstream step remaining unresolved	Minigenome/polymerase luciferase assay; authentic IAV infection; strand-specific vRNA/mRNA RT-qPCR; CRL4-factor overexpression and siRNA depletion; ubiquitin-linkage and PB2 lysine-site mutant/recombinant-virus analysis; polymerase and viral-RNA readouts showed no significant effect following host-factor depletion.

Note: Validation methods were classified conservatively. Reporter-virus luciferase readouts were not counted as minigenome/polymerase assays unless a PB1-PB2-PA-NP reconstitution system with a vRNA-like reporter was reported. Viral RNA readouts were listed only when direct RNA measurements were reported; RNA measurements in reconstituted vRNP or plasmid-expression systems are labeled as indirect where applicable.

**Table A2:** Evidence-tiered map of site-resolved ubiquitin/UBL modifications of IAV FluPol and NP.

Protein	Site(s) or Landscape	Evidence Status and Infection Context	Conservation	Structural Context and Functional Consequence	Reference
PB1	15 K-epsilon-GG sites	Same landscape study; seven sites were surface-exposed and the remainder were buried or covered in the trimeric complex.	As above; site-specific values were directly reported for K578.	Sites occur in the polymerase core, nucleotide triphosphate (NTP) tunnel, and thumb region; most require independent mechanistic validation.	[20]
PB1	K229, K347, K471	K-epsilon-GG mapping plus A/R mutational analysis.	Not separately reported.	Polymerase catalytic cavity; mutations impaired polymerase activity and virus rescue, but this does not by itself prove a ubiquitin-specific mechanism.	[20]
PB1	K360	K-epsilon-GG mapping plus A/R mutational analysis.	Not separately reported.	Polymerase core; substitution altered RNA-synthesis readouts, while the responsible modifier remains unassigned.	[20]
PB1	K480	K-epsilon-GG mapping.	Not separately reported.	Rim of the NTP tunnel; functional consequence remains uncharacterized.	[20]
PB1	K578	Direct ubiquitin acceptor-site evidence, mutational analysis, and reverse-genetics experiments; the responsible E3 ligase is unknown.	K is present in 98.82% of human, 99.26% of avian, and 75.18% of swine sequences analyzed in the source study.	PB1 thumb domain; affects NP binding, polymerase dimerization, and vRNA replication. The proposed PB1-PB2 allosteric mechanism remains model-supported.	[20]
PB1	K635	K-epsilon-GG mapping plus A/R mutational analysis.	Not separately reported.	Thumb domain; mutation increased polymerase activity, consistent with a possible restrictive effect of the modification.	[20]
PB2	22 K-epsilon-GG sites	Landscape-level detection in A/WSN/1933-infected A549 cells (5 h post-infection); modifier identity is unresolved for most sites.	Most of the 59 polymerase sites were reported as highly conserved across human, swine, and avian isolates; individual values are not reproduced here.	Sites cluster in the N-terminus/N2 region and the C-terminal 627-NLS region; most individual functions remain unknown.	[20]
PB2	K157	K-epsilon-GG mapping plus alanine/arginine (A/R) mutational analysis in the polymerase/reverse-genetics screen.	Not separately reported.	mRNA exit tunnel; mutation increased polymerase activity, consistent with a possible restrictive effect of the modification.	[20]
PB2	K482, K752	K29-linked PB2 ubiquitination associated with CRL4-DCAF12L1; infection and recombinant-virus evidence supports a proviral role.	Not separately reported.	Non-degradative PB2 modification; the exact downstream step remains unresolved.	[20,53,54]
PB2	K660, K663	K29-linked PB2 ubiquitination associated with CRL4-DCAF11; infection and recombinant-virus evidence supports a proviral role.	Not separately reported.	Non-degradative PB2 modification; the exact downstream step remains unresolved.	[53,54]
PB2	K617	K-epsilon-GG mapping plus A/R mutational analysis.	Not separately reported.	Putative NP-binding region; mutation indicates a possible proviral contribution, but the modifier is not assigned.	[20]
PB2	K699	K-epsilon-GG mapping; this residue was also reported as a NEDD8 acceptor site.	Not separately reported.	Nuclear transport-associated PB2 region; the ubiquitin-specific role remains unresolved.	[20,23]
PA	22 K-epsilon-GG sites	Same landscape study; seven sites map to the endonuclease domain.	Most sites reported as conserved; site-specific values are not reproduced here.	Sites span endonuclease, linker, C-terminal dimerization, and RNA polymerase II interaction regions; most remain uncharacterized.	[20]
PA	K22	K-epsilon-GG mapping plus A/R mutational analysis.	Not separately reported.	Hinge at the endonuclease/PB1 interface; substitutions had divergent effects on polymerase activity.	[20]
PA	K102, K104	K-epsilon-GG mapping.	Not separately reported.	Face the PB2 cap-binding domain in the transcriptase conformation and have been linked to the capped mRNA-primer interaction; functional modification effect remains unresolved.	[20]
PA	K134	K-epsilon-GG mapping plus A/R mutational analysis.	Not separately reported.	Endonuclease catalytic region; substitutions impaired polymerase activity and virus rescue, but modifier-specific causality is unresolved.	[20]
PA	K536	K-epsilon-GG mapping plus reverse-genetics analysis.	Not separately reported.	Putative RNA-template binding groove; mutant viruses rapidly reverted, indicating strong selection for lysine but not a defined modifier mechanism.	[20]
PA	K609	K48-linked ubiquitination by CRL4B-DCAF7 in infection, with genetic and in vivo evidence.	Not separately reported.	PA degradation restricts polymerase activity and IAV replication.	[56]
PA	K615	K-epsilon-GG mapping plus A/R mutational analysis.	Not separately reported.	Functional contribution to virus generation was observed, but the modifier and mechanism are unresolved.	[20]
NP	K184, K227, K273	CNOT4-associated NP monoubiquitination with mutational and polymerase/replication assays; USP11 removes NP-K184 monoubiquitination.	Not systematically reported across host lineages.	Within or near the RNA-binding groove; supports vRNP activity and viral RNA replication in the tested system.	[62,63]
NP	K351	Pirh2-mediated short-chain ubiquitination with host-factor perturbation and rescued-virus evidence.	K in 99.18% of human isolates; R in 95.3% of avian isolates in the reported dataset.	NP-PB2 interaction region; modification impairs vRNP formation and restricts replication. The phenotype is strain- and residue-dependent.	[64]

Note: K-epsilon-GG evidence identifies a lysine modified by ubiquitin or a ubiquitin-like modifier, but it does not by itself identify the attached modifier, ubiquitin linkage, E3 ligase, or functional consequence. Rows are grouped by viral protein in the order PB1, PB2, PA, and NP. The landscape rows summarize the complete 59-site polymerase landscape reported in infected cells; the remaining rows list published positions for which structural or functional context is available.
